# Therapeutic potential of stem cell-derived extracellular vesicles in osteoarthritis: preclinical study findings

**DOI:** 10.1186/s42826-020-00043-3

**Published:** 2020-04-15

**Authors:** Ki Hoon Kim, Jeong Hyun Jo, Hye Jin Cho, Tae Sub Park, Tae Min Kim

**Affiliations:** 1 Graduate School of International Agricultural Technology, Pyeongchang, South Korea; 2 Institutes of Green-Bio Science and Technology, Seoul National University, Pyeongchang, Gangwon-do 25354 South Korea

**Keywords:** EVs (extracellular vesicles), MSCs (Mesenchymal stem cells), OA (osteoarthritis)

## Abstract

Extracellular vesicles (EVs) are nano-sized particles secreted by almost all cell types, and they mediate various biological processes via cell-to-cell communication. Compared with parental cells for therapeutic purposes, stem cell-derived EVs have several advantages such as reduced risk of rejection, less oncogenic potential, ease of long-term storage, lower chance of thromboembolism, and readiness for immediate use. Recent studies have demonstrated that EVs from stem cells, mostly from mesenchymal stem cells (MSCs) from various tissues, have anti-inflammatory, anti-oxidative, anti-apoptotic, and proliferative role in injured organs including osteoarthritic lesions. Herein, we provide a review about the up-to-date studies in preclinical application of stem cell-derived EVs in osteoarthritis animal arthritis models.

## Introduction

Among joint diseases, osteoarthritis (OA) is one of the most severe types of arthritis that is caused by loss of joint cartilage and bone [1]. Mostly, the articular damage is due to loss of self-repair capability of injured cartilage caused by mechanical stress, e.g., sudden or unadjusted movements, mechanical injury, excess weight, loss of muscle strength supporting joint, and damage in peripheral nerves [2]. Also, it is still under debate whether exercise increases the risk of osteoarthritis in the knee [3].

### Osteoarthritis: its pathophysiology

**Fig. 1:**
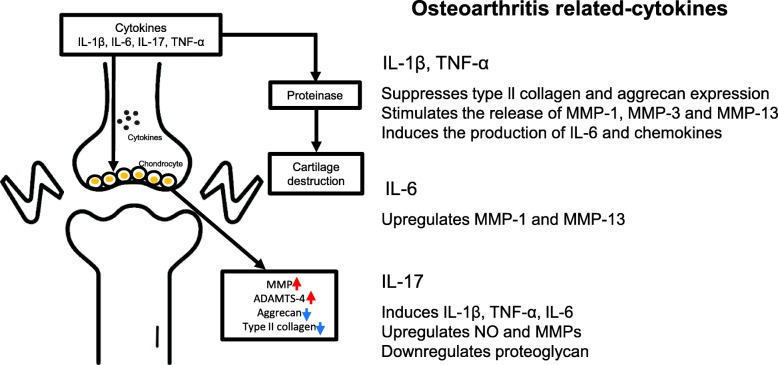
The role of proinflammatory cytokines in the pathophysiology of OA. The role of proinflammatory cytokines, including IL-1β, TNF-α, IL-6 and IL-17, are elevated in OA. These cytokines contribute to the pathogenesis of OA through several mechanisms including downregulation and upregulation of inflammatory responses. Abbreviations: ADAMTS: a disintegrin-like and metalloproteinase with thrombospondin motifs; IL: interleukin; MMP: matrix metalloproteinase; NO: nitric oxide; TNF: tumor necrosis factor

So far various soluble mediators have been reported to be involved in the progression of OA. Readers are referred to other reviews on the detailed role of the role of pro-inflammatory (IL-1β, TNF-α, IL-6, IL-17) (Fig. 1) and anti-inflammatory cytokines that are involved in OA pathogenesis (IL-4, IL-10, IL-13) [4, 5]. For example, an elevated level of IL-1β and TNF-α was found in OA synovial fluid, synovial membrane, and subchondral bone cartilage [6]. Mechanistically, these cytokines down-regulated the synthesis extracellular matrix (ECM) component by inhibiting anabolic activities of chondrocytes. Another study showed that IL-1β reduces the expression of the type II collagen, which is a major ECM component constituting the cartilaginous tissues in several animal species [7, 8]. Also, the expression of Aggrecan, which is one of the major components of the cartilage, was found to be decreased by IL-1β treatment in chondrocytes and cartilage [9]. Indirectly, IL-1β and TNF-α stimulate chondrocyte to produce a proteolytic enzyme such as matrix metalloproteinases (MMPs), including MMP-1 (interstitial collagenase), MMP-3 (stromelysin 1), MMP-13 (collagenase 3) [10–12]. In addition, ADAMTS (a disintegrin-like and metalloproteinase with thrombospondin motifs) of is also one of the major players in cartilage degradation in OA. It was reported that the expression of ADAMTS-4 can be induced by IL-1β and TNF-α, while the expression of ADAMTS-5 was not affected [13]. In contrast, subsequent study has shown that IL-1β induced its mRNA expression in rabbit nucleus [14]. Other than these proteases, miR30a was also shown to play an important role in controlling ADAMTS-5 expression that was caused by IL-1β [15]. Also, IL-1β and TNF-α induce the generation of inflammatory cytokines such as IL-6 [14] and IL-8 [16], monocyte chemoattractant protein 1 (MCP1) [17] and CC-chemokine ligand 5 (CCL5) [18], all of which are well-reported players in sustaining tissue inflammation. IL-6 exists at a low concentration level in normal chondrocyte. However, its concentration in sera and chondrocytes is increased in osteoarthritic condition, after which it causes the increases in IL-1β and TGF-β, which in turn they promoted the production of IL-6 [19, 20]. Studies also demonstrated that IL-6 stimulates the expression of MMP-1 and MMP-13 in bovine and humans (cell type) [21, 22], and IL-6 reduced the expression of type II collagen (cell type) [23]. Other studies showed that the expression of IL-17 is upregulated by IL-1β, TNF-α and IL-6, after which IL-17 upregulated NO and MMPs production [24]. In addition, IL-17 led to a reduced expression of proteoglycan [25].

### Current treatment method for OA

**Fig. 2:**
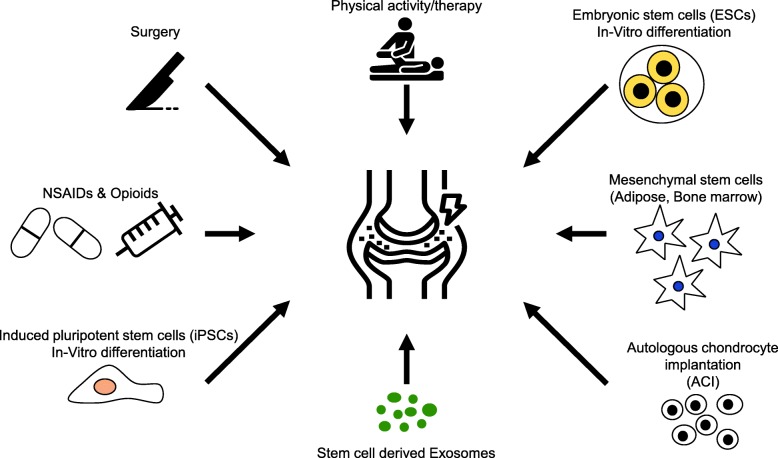
Current treatment methods and the potential for cell-based treatment methods in OA. Abbreviations: ACI: autologous chondrocyte implantation; ESCs: embryonic stem cells; iPSCs: induced pluripotent stem cells; NSAIDs: non-steroidal anti-inflammatory drugs

Depending on the disease status, clinical protocols can be classified into surgical method, using NSAIDs (Non-steroidal anti-inflammatory drugs), via physical therapy, opioids, or intra-articular injection of hyaluronic acid (Fig. 2). Although NSAIDs have been commonly used for relieving inflammation due to their analgesic and anti-inflammatory effect, side effects such as the organ toxicity (e.g., liver and kidney) have been critical. In particular, using NSAIDs for a long-term or repeated time can lead to gastrointestinal tract hemorrhage [26–28]. Thus, other alternatives, i.e., cellular therapies using autologous or allogenic origins are now becoming recognized as save and effective option. Also, application of induced pluripotent stem cell (iPSC)-derived chondrocytes may be another choice depending on the regulation and safety guidelines [29]. Although several protocols are currently available for clinical purposes [30], cell-based therapy inherently possess the risk of immune rejection and tumor formation in vivo [31, 32]. Accordingly, application of extracellular vesicles, which can be obtained from desired cell types during culture, would be an ideal cell-free strategy that can solve the problems that can be raised upon implementing cell therapy [29, 33, 34].

### Isolation methods of EVs

Currently, various protocols are being used to isolate EVs. Classically, ultracentrifugation is recognized as the most standardized method for isolating a large scale of EVs. Other methods include immunoaffinity isolation (magnetic bead isolation), tangential flow filtration, size exclusion chromatography, and polymer precipitation [35–38]. Ultracentrifugation can be modified or further optimized, such as applying density gradient force during ultracentrifugation for harvesting EVs with an enhanced purity. To obtain EVs with higher purity in a large amount, tangential flow filtration method has been developed. This technique enables the removal of cell debris and unnecessary biomolecules by filtering the cell culture supernatant using sterile hollow fiber polyethersulfone membrane [39]. This system may be ideal for producing in an industrial (20–50 l) or laboratory scale (e.g.,300 ml) [37, 40]. Size exclusion (chromatography) method is based on physical barriers, for example filters or chromatographs. Ideally, this technique enables removing many contaminating elements such as albumin or lipoproteins. Depending on the size of EVs that are of interest, a range of different pore sizes (0.8 or 0.2 μm) may be used [41, 42]. Finally, polymeric precipitation method is relatively easy and quick, and optimal for enriching EVs for small-scale experimental purposes. Precipitation mixtures are incubated with culture medium, and low speed centrifugation is used to concentrate EVs [43]. There is, however, a study showing the quality of RNA extracted from polymeric precipitation may not be optimal [44].

### Characterization of EVs

EVs are collective term for heterogeneous nano-sized lipid-bilayerd membrane vesicles having 30-2000 nm diameter. Importantly, EVs play essential role in intercellular communications due to a large variety of biologically active signaling molecules within EVs, including RNA species (messenger RNA and small RNA), proteins, enzymes, lipids and DNA fragments [45]. So far various characterization methods are available. TEM (Transmission electron microscopy) and SEM (Scanning electron microscopy) are usually used for verifying their cup- or round- shape [46–49]. TEM is more commonly used than SEM [50, 51]. The diameter size as well as their size distribution can be measured by NTA (nanoparticle tracking analyzer) [49, 52]. Finally, the presence of EV-specific markers (CD9. CD63, CD81, TSG101, and Alix) [48, 49] can be examined by immunoblotting or flow cytometry.

### Preclinical studies

EVs contain a wide spectrum of biomolecules including proteins, lipids, nucleic acids (DNAs, RNAs, small RNAs). Together with the notion that EVs are a natural player of cell-cell interaction in multicellular organisms, studies have focused on strengthening their specific function [53]. For example, miR-140-5p-overexpressing synovial MSC-derived exosomes led to an enhancement of chondrocyte proliferation and migration, and prevented OA in a medial meniscus OA rat model [54]. Other study showed that human embryonic MSC-derived exosomes injected in medial meniscus OA mouse model improved the synthesis of cartilage regeneration [55]. Another study compared the therapeutic efficacy between EVs from iPSC derived - mesenchymal stem-like cells and synovial membrane-derived MSC in Collagenase-induced mouse OA model, and showed that EVs from iPSC derived MSC was better in reducing OA progression [56]. Similarly, in a rat model of osteochondral defect, EVs derived from human embryonic mesenchymal stems was able to reduce the disease progression [57]. Several mechanistic studies also showed that EVs from MSCs mediate cartilage repair by enhancing proliferation, attenuating apoptosis, modulating immune reactivity. For example, treatment of MSC-derived exosomes led to an enhanced activity of AKT and ERK signaling in cultured chondrocytes in vitro, and an increased infiltration of CD163+ regenerative M2 macrophages over CD86+ M1 macrophages was found in the osteochondral tissue in Surgical defect created on the model [34]. In addition, EVs from mouse BM-MSCs showed a therapeutic effect in collagenase induced arthritis model, as shown by Protection from osteoarthritis damage and a reduction of apoptotic cells injected in mouse chondrocyte, with a significant improvement cartilage generation. Finally, EV treatment was able to reduce osteophyte formation in a mice model of OA [58]. In an OA model created by making a rounded trephine grooves osteochondral model in dogs (3 mm diameter, 1 mm depth), administration of mouse bone marrow MSC-derived EV led to a marked regeneration of cartilage and restoration of chondral tissue [59]. Also, it was shown that WNT5A expression was inhibited by miR-92a-3p delivery by exosomes, which led to an inhibition of cartilage degradation [60]. In a collagenase induced arthritis model in mice, EVs from mouse BM-MSCs inhibited T lymphocyte proliferation in a dose-dependent manner, and also decreased the percentages of CD4 and CD8 subsets. Also, fewer plasmablasts and more Breg-like cell in lymph nodes was found [61].

miRNAs are one of the major biological cargoes in EVs from parental cells, and it was shown that miR-100-5p was enriched in the exosomes derived from human Infrapatellar fat mesenchymal stem cells. Upon being injected intra-articular into OA mice induced by destabilization of the medial meniscus, the OA progression was dramatically attenuated, as shown by the reduction of articular damage and amelioration of gait abnormality. Molecular study also demonstrated that miR-100-5p inhibited mTOR/ autophagy pathway [62]. Another study demonstrated that exosomes from miR-92a-3p-overexpressing BM-MSCs was able to promote the chondrocyte proliferation, and upregulated several matrix genes (Aggrecan, Col2A1, Sox9) and decreased a subset of other matrix genes (Col2A10, Runx2, MMP13, Wnt5A).

Another study showed that EVs from human amniotic fluid stem cells has therapeutic effect in MIA (Monoiodoacetate)-induced OA model in rats, as demonstrated by an enhanced pain tolerance and improved histological score. After 3 weeks of EV treatment, rat cartilage restoration with good surface regularity and with the characteristic of hyaline cartilage was shown. Moreover, markers of resolving marcrophages (CD163, arginase 1, and TGFβ) were significantly increased after EV treatment [63].

Collectively, EVs from various stem cells alleviated the disease progression, as supported by results of tissue histology as well as inflammatory cytokine profiles in various preclinical OA models. We have provided a detailed list of studies that have attempted to use EVs from various parental cell types in OA animal models (Table 1).

**Table 1:** The therapeutic effect of extracellular vesicles in preclinical osteoarthritis models

Cell types	EV characterization methods	Main cargo	Animal models	Results	References
Human embryonic stem cells	WB (CD81, TSG101, ALIX)	Not mentioned	Surgical defect on trochlear grooves of the distal femurs osteochondral model in rats (1.5 mm diameter, 1 mm depth)	Hyaline cartilage formation characterized by uniform distribution of high amounts of glycosamninoglycan (GAG) and type II collagen, and low amount of type I collagen	[57]
Synovial mesenchymal stem cells (SMSCs)	WB (CD9, CD63, CD81, ALIX)	miR-140-5p	OA induced by medial meniscus surgery in rats	Prevention of early OA and prevented the severe damage to knee articular cartilage with increased type II collagen deposition	[54]
H1 human ES cell line	WB (CD9, CD63)	Not mentioned	OA induced by medial meniscus surgery in mice	Milder OA pathology such as roughened articular surface fibrillations below the superficial layer and some loss of lamina, with increased collagen II and decreased ADAMTS5 expression.	[55]
iPSCs- and synovial membrane- derived MSCs	WB (CD9, CD63, TSG101)	CD9, CD63 and TSG101	Collagen-induced OA model in mice	Re-formation of hyaline features with a smooth cartilage surface, regular cellular organization, and normal proteoglycan content. Decreased collagen I in animals treated with iMSC-Exos or SMMSC-Exos	[56]
E1-MYC 16.3 human ESC- derived mesenchymal stem cells	WB (CD81, TSG101, ALIX)	Not mentioned	Surgical defect created on the trochlear grooves of the distal femurs osteochondral model in rats (1.5 mm diameter, 1 mm depth)	Tissue regeneration deposition of s-GAG and type II collagen. Improved surface regularity and integration with the host cartilage	[34]
Murine Bone marrow mesenchymal stem cells	FC (CD9, CD29, CD44, CD81, SCA-1), NTA	Not mentioned	Collagenase induced OA model in mice	Protection against osteoarthritic damages via anti-apoptotic role	[58]
Bone marrow mesenchymal stem cells	FC (CD63, CD44, CD73)	Not memtioned	Surgical defect created on rounded trephine grooves osteochondral model in dogs (3 mm diameter, 1 mm depth)	Marked regeneration of cartilage tissues	[59]
Human Bone marrow mesenchymal stem cells	NTA TEM (Transmission electron microscope)	miR-92a-3p	Collagenase induced OA model in mice	The proliferation of chondrocytes Increased the matrix gene expression in MSCs and PHCs. miR-92-3p-mediated inhibition of WNT5A was shown.	[60]
Mice Bone marrow mesenchymal stem cells	NTA, TEM, FC (CD9, CD29, CD44, CD81, SCA-1)	Not memtioned	Collagenase induced OA model in mice	Induced a fewer plasmablasts and more Breg-like cells in lymph nodes. Induced an anti-inflammatory role on T and B cells	[61]
Human Infrapatellar fat mesenchymal stem cells	NTA, TEM, WB (CD9, CD63, CD81)	miR-100-5p	Cutting the medial meniscus OA model in mice	Protected articular cartilage from damage and ameliorated gait abnormality miR100-5p-mediated inhibition of mTOR-autopahgy pathway was shown	[62]
Human Amniotic fluid stem cells	WB (CD9, CD63, CD81, Rab5, HGF, TGF- β, IDO)	Not mentioned	MIA induced OA in rats	Enhanced pain tolerance and improved histological scores. Restoration of cartilage with surface regularity and the characteristic of hyaline cartilage.	[63]

Abbreviations: *ADAMTS* a disintegrin-like and metalloproteinase with thrombospondin motifs; *ESCs*: embryonic stem cells; *FC*: flow cytometry; *HGF*: hepatocyte growth factor; *IDO*: indoleamine-pyrrole 2,3-dioxygenase; *iPSCs*: induced pluripotent stem cells; *MIA*: monoiodoacetate; MSCs: mesenchymal stem cells; mTOR: mammalian target of rapamycin; *NTA*: nanoparticle tracking analyzer; *PHC*: primary human chondrocyte; *S-GAG*: sulfate-glycosaminoglycan; *TEM*, transmission electron microscopy; TGF: transforming growth factor; *TSG101*: tumor susceptibility gene 101; *WB*: western blot

## Conclusion

EV carry out many different functions in organisms that include repair of tissue injuries, regulation of immune response, and inhibition of inflammation. The improvement in arthritic pathologies by MSCs is mostly due to cell-to-cell direct interaction and also by secretion of various soluble mediators. This review has presented MSC derived EVs as a cell-free treatment of joint damage and OA. It is currently accepted that the biological contents of EVs may significantly differ from those from parental cells, thus more extensive characterization of the membrane bound or luminal cargoes needed to further application of these unique nano-sized particles for therapeutic uses.

## Data Availability

Not applicable.

## Abbreviations

ACI: Autologous chondrocyte implantation
ADAMTS: A disintegrin-like and metalloproteinase with thrombospondin motifs
BM: Bone marrow
CCL5: CC-chemokine ligand 5
CD: Cluster of differentiation
Col2A1: Collagen type II alpha 2
Col2A10: Collagen type II alpha 10
ECM: Extracellular matrix
ESCs: Embryonic stem cells
EVs: Extracellular vesicles
FC: Flow cytometry
HGF: Hepatocyte growth factor
IDO: Indoleamine-pyrrole 2,3-dioxygenase
IL: Interleukin
iPSCs: Induced pluripotent stem cells
MCP1: Monocyte chemoattractant protein 1
MIA: Monoiodoacetate
MMP: Matrix metalloproteinase
MSCs: Mesenchymal stem cells
mTOR: Mammalian target of rapamycin
NO: Nitric oxide
NSAIDs: Non-steroidal anti-inflammatory drugs
NTA: Nanoparticle tracking analyzer
OA: Osteoarthritis
PHC: Primary human chondrocyte
Runx2: Runt-related transcription factor 2
SEM: Scanning electron microscopy
S-GAG: Sulfate-glycosaminoglycan
TEM: Transmission electron microscopy
TGF: Transforming growth factor
TNF: Tumor necrosis factor
TSG101: Tumor susceptibility gene 101
WB: Western blot
